# A Survey of Pediatric CT Protocols and Radiation Doses in South Korean Hospitals to Optimize the Radiation Dose for Pediatric CT Scanning: Erratum

**DOI:** 10.1097/01.md.0000480959.79399.3d

**Published:** 2016-02-12

In the article “A Survey of Pediatric CT Protocols and Radiation Doses in South Korean Hospitals to Optimize the Radiation Dose for Pediatric CT Scanning”,^1^ which appeared in Volume 94, Issue 50 of *Medicine*, the original Figures 1–7 have been replaced with higher-quality images. The article has since been corrected online.
